# Asynchronous group learning in learn from the learner approach: A Learning Object That Enhances and Facilitates Distance Self and Shared Learning

**DOI:** 10.1016/j.amsu.2021.102535

**Published:** 2021-07-02

**Authors:** Faiz Tuma, Jafar Aljazeeri

**Affiliations:** Central Michigan University College of Medicine, USA; University of Pittsburgh Medical Center (UPMC), USA

**Keywords:** Medical education, Asynchronous learning

## Abstract

Learning with the learner in an asynchronous group learning approach is a promising method of education that provides a rich, interactive, and socially mediated education. As online learning became more prevalent, and more users are adopting this approach, innovative and theory-based educational activities became necessary. In this article, we introduce and describe a novel form of asynchronous, interactive, and socializing educational activity using educational technology.

The educational session is based on a small group learning activity that is made available for all learners anywhere and anytime. The approach avoids the trap of using educational technology for mere simulation of in-person learning. Based on learning theories, learning with the learner enhances interactive, self-directed, experiential, and social learning. Future development and enhancement with ongoing discussions through online chat platforms open the door for the continuous evolution of the concept.

## Highlights

• Educational technology has evolved from basic use to provide self-directed learning opportunities for learners.
• Learning from learners offers a stimulating, socially interactive, and entertaining educational alternative approach.
• It provides a better alternative to the classical video lectures or other unidirectional delivery of information activities.
• It is a practical, easy to structure with a low-cost approach that accommodates how students like to learn.

## 1 Introduction

Medical education faces continuous challenges in the process of facilitating learning and training. Learning is a complex, multifactorial, and evolving process. There are still challenges that require improvement and innovation to identify better opportunities. Therefore, efforts to accommodate learners’ needs continue to find more efficient learning approaches and tools to acclimatize the growing demands. One of these approaches is facilitating interactive learning. Interactive and group learning approaches have been widely used in medical education to enhance learning. Sharing facilitates learning in various ways. Sharing ideas, perceptions, understandings, ways of thinking, and other higher mental skills and making this sharing available for all learners is a valuable venue for better education. Group studying enhances interactive learning and sharing. However, enhancing interaction and transforming educational activities into interactive sessions is often challenging for educators.

The traditional group study takes place in a face-to-face fashion. Nonetheless, with the advanced technology and communication, distant asynchronous studying in group fashion became more possible and practical, especially during the COVID-19 pandemic. Educational technology instruction can be integrated with group learning activities and experiential learning to enhance learning significantly [1]. The aim of this article is to introduce and describe a new approach to interactive, asynchronous group learning using simple educational technology. Learners can share their experiences to enrich the educational process and exchange learning skills asynchronously and practically.

## 2 Technology provides the tools and concept

Educational technology provides more than just storage of digitalized learning materials or information in books and journals. Technology provides the communication, mobility, and instructional designs to facilitate learning. Educational technology in medical education has evolved from basic use of resources to advanced web-based multimedia instruction, to provide self-directed learning opportunities for learners [2]. Enhancing self-directed learning through distance education or e-learning opens a broad concept of learning with minimal synchronous instructor involvement.

E-learning provides a practical and accessible technology learning tool to overcome various challenges and limitations in learning activities among various health care courses [3]. Furthermore, the blended learning approach to combine e-learning with face-to-face interactions to improve interprofessional competencies has been increasingly used [2]. Blended learning refers to using computer-mediated learning in-person interactions [2]. Blending virtual and in-person simulations can optimize learning efficiency [4]. Various educational activities such as didactic teaching, discussions, and self-directed learning can be achieved with e-learning. While other activities such as simulation of psychomotor skills may require in-person learning. Hence, blended learning provides a promising alternative approach for medical education because it combines the advantages of both traditional learning and e-learning [5].

## 3 Sharing learning

Sharing learning experiences in the asynchronous type of group learning where learners can learn from other learners' experiences can be structured to provide important learning objects. Educational technology facilitates video recording of a small group learning activity focusing on learners’ interactions, various inputs, and conclusions. These interactions that involve learning skills, thinking talents, analysis abilities, and conclusions competencies make the core elements of the recorded activity.

The educational activity is structured by educators for a small group independent learning. Two or 3 students use the educational activity to learn in an interactive group setting involving enhanced and diverse interactions. This learning activity that includes all interactions is recorded and made available to other learners in the form of vodcast, podcast, or other digital media versions. other learners can use this recorded experience as an adjunct or as a frame to learn the same topic. Using this recorded activity by other learners to study the same topic will enrich the learning experience with all the interactions of the original participants. It simulates studying with a group in a distance or blended learning that is available anytime and anywhere.

The next step in developing this approach is building ongoing discussion and comments from all viewing learners. A chat platform that contains the originally recorded activity can be made available for all the comments and textual input of other learners or educators. This ongoing input and asynchronous discussion will augment the sharing process, provides review and feedback to the ideas, and stimulates considering alternatives.

## 4 Outcomes

The advantages of this learning approach include: 1) creating a unique educational activity that records the valuable learners' interactions and contributions and making it available to all learners at anytime and anywhere; 2) sharing the diverse and valuable learning skills, analysis, and critical thinking; 3) facilitating self-learning in a simulated socially-rich environment, and 4) transforming unidirectional teaching to shared learning and semi-interactive education.

Education has already been more influenced by social interaction [6]. Younger generation learners commonly use social interactions in a ‘digital natives’ style [6]. This learning approach exemplifies a simulation of the social learning environment. It allows learners to compare themselves to others, facilitates understanding, widens the scope of critical thinking, and enhances attentiveness in a social environment. It also provides a valuable company for learners to stimulate and enhance learning. Group learning enhances active and self-directed learning, reflection upon learning activities, self-regulatory skills, testing and comparing own thinking, hypothesis, deep learning and higher-order activities, adult style of learning, and acceptance of responsibility for own progress [7]. It improves transferable skills such as organizational, and time management skills, leadership, prioritization, and problem-solving [8].

## 5 Conclusions

Learning from learners can provide rich, stimulating, facilitating, social, interactive, and entertaining educational activities with easy structure and low cost. It provides a better alternative to the classical video lectures or other unidirectional delivery of information activities. It is based on how students like to learn conveniently. Evaluating and validating the use of this adjunctive method of learning is important. Various settings can be structured to assess the method and to develop it further depending on the feedback.

## Provenance and peer review

Not commissioned, Editor reviewed.

## Ethical approval

This a perspective in medical education. No ethical approval was needed.

## Sources of funding

There is no source of funding for this study.

## Author contribution

Faiz Tuma conceived the main ideas of the article and wrote the first draft of the manuscript. Jafar Aljazeeri critically revised the manuscript and provided additional points. The final version approved by both authors.

## Trial registry number

1. Name of the registry: N/A
2. Unique Identifying number or registration ID: N/A
3. Hyperlink to your specific registration (must be publicly accessible and will be checked): N/A

## Guarantor

Faiz Tuma, MD, MME, MDE, EdS, FACS, FRCSC.

Associate Professor, Department of Surgery.

Central Michigan University College of Medicine.

912 S. Washington Avenue, Suite #1 Saginaw, MI 48601-2578.

Phone: 989- 790–1001. Fax: 989-790-1002.

E-mail: faiz.tuma@cmich.edu.

## Consent

N/A.

## Declaration of competing interest

All the authors have no conflict of interest related to this study to disclose.
